# An Emerging Allee Effect Is Critical for Tumor Initiation and Persistence

**DOI:** 10.1371/journal.pcbi.1004366

**Published:** 2015-09-03

**Authors:** Katrin Böttger, Haralambos Hatzikirou, Anja Voss-Böhme, Elisabetta Ada Cavalcanti-Adam, Miguel A. Herrero, Andreas Deutsch

**Affiliations:** 1 Center for Information Services and High Performance Computing, Technische Universität Dresden, Dresden, Germany; 2 Center for Advancing Electronics, Technische Universität Dresden, Dresden, Germany; 3 Hochschule für Technik und Wirtschaft Dresden, Dresden, Germany; 4 Department of Biophysical Chemistry, Institute of Physical Chemistry, University of Heidelberg, Heidelberg, Germany; 5 Max Planck Institute for Intelligent Systems, Stuttgart, Germany; 6 Departamento de Matemática Aplicada, Facultad de Matemáticas, Universidad Complutense, Madrid, Spain; University of Notre Dame, UNITED STATES

## Abstract

Tumor cells develop different strategies to cope with changing microenvironmental conditions. A prominent example is the adaptive phenotypic switching between cell migration and proliferation. While it has been shown that the migration-proliferation plasticity influences tumor spread, it remains unclear how this particular phenotypic plasticity affects overall tumor growth, in particular initiation and persistence. To address this problem, we formulate and study a mathematical model of spatio-temporal tumor dynamics which incorporates the microenvironmental influence through a local cell density dependence. Our analysis reveals that two dynamic regimes can be distinguished. If cell motility is allowed to increase with local cell density, any tumor cell population will persist in time, irrespective of its initial size. On the contrary, if cell motility is assumed to decrease with respect to local cell density, any tumor population below a certain size threshold will eventually extinguish, a fact usually termed as Allee effect in ecology. These results suggest that strategies aimed at modulating migration are worth to be explored as alternatives to those mainly focused at keeping tumor proliferation under control.

## Author Summary

Controlling tumor growth remains a major medical challenge. Current clinical therapies focus on strategies to reduce tumor cell proliferation. However, during tumor progression, tumor cells may switch between proliferative and migratory behaviors, thereby allowing adaptation to microenvironmental changes that result in variations in local tumor cell density. We herein explore by means of a mathematical model the impact of migration-proliferation plasticity on tumor initiation and persistence. Our work suggests that small tumors can become extinct solely by their intrinsic cell population dynamics if cell motility decreases along with local cell density. In contrast, if cell motility increases with cell density, the tumor inevitably grows. Our model suggests that the regulation of cell migration plays a key role in tumor growth as a whole, making this feature a potential target for clinical studies.

## Introduction

Tumor cells possess a remarkable phenotypic plasticity that allows for adaptation to changing microenvironmental conditions [1, 2]. Well-known examples are the epithelial-mesenchymal transition [3, 4] and the shift from ATP generation through oxidative phosphorylation to an anaerobic, glycolytic metabolism, often referred to as the Warburg effect [5]. A further example is phenotypic plasticity with respect to cell proliferation and migration [6], a phenomenon related to the “go-or-grow” mechanism. Such a migration-proliferation dichotomy has been observed for non-neoplastic cells [7, 8] as well as in the course of tumor development [9–11]. The precise molecular mechanisms underlying this dichotomy remain poorly understood. It has been suggested that the switch between migrating and proliferative phenotypes is dependent on the cells’ microenvironment such as growth factor gradients [7], properties of the extracellular matrix [12] or altered energy availability [13]. In this context, several mathematical models have shown that the migration-proliferation plasticity has a major impact on tumor spread [14–19]. There is a growing body of evidence which suggests that local cell density is correlated with gradients of nutrients, secreted factors, oxygen or toxic metabolites [20, 21]. Hence, local cell density can be considered as a core factor for analyzing the dependence of the switch on the tumor microenvironment. However, while the consequences of density-dependent migration-proliferation plasticity on local tumor spread, as an essential feature of tumor invasion, have been explored already [14, 18, 19], the potential effects of this type of plasticity on tumor initiation and persistence have not been investigated so far.

In this work we point out some aspects of the phenotypic plasticity between migratory and proliferative phenotypes for tumor growth that have been unnoticed so far. To do this, we make use of a suitable mathematical model to be described below. We note in this context that mathematical models have proven successful for analyzing various aspects of tumor dynamics, see for example [22–24]. More precisely, we formulate and study a model that allows to derive the overall tumor cell population dynamics as an emergent property resulting from individual cell behavior. This is achieved by means of a cellular automaton model which extends model rules used in previous studies, where the impact of a migration-proliferation dichotomy has been investigated with a clear focus on tumor invasion [19, 25, 26]. Here, for the first time, the influence of a density-dependent migration-proliferation plasticity on tumor growth and persistence is studied. In our model, the switch between migratory and proliferative phenotypes is made explicitly dependent on the microenvironment, in particular on cell density. Analysis of our model reveals that two dynamically different regimes can be distinguished. If cell motility increases with local cell density, even a small initial tumor population will always grow. This regime can be associated to a biological situation where contact inhibition of cell migration (CIM) is downregulated. On the contrary, if cell motility decreases with local cell density, which is the case if CIM is present, tumor colonies which are small enough can be driven to extinction by the intrinsic cell population dynamics. We unveil that this behavior is a consequence of negative growth rates emerging at low densities, a phenomenon called Allee effect in ecology [27]. Accordingly, controlling tumor cell migration would have significant consequences not just for tumor dissemination, but also for overall tumor progression. In fact, our work predicts that tumors can potentially be driven to extinction if CIM is externally enhanced. However, loss of such type of inhibition will invariably lead to tumor persistence.

## Materials and Methods

### Model definition

We develop a stochastic, spatio-temporal cell-based model to study the effects of density-dependent phenotypic plasticity. In this way we account for single cell behavior that depends on the local, spatial microenvironment and for microscopic fluctuations which reflect cellular and microenvironmental heterogeneity. To do that, a discrete model, namely a lattice-gas cellular automaton (LGCA) is defined. LGCA models are well-suited to model cell-cell interaction and cell migration [28–30].

The LGCA model is described on a discrete *d*-dimensional regular lattice *ℒ* with periodic boundary conditions. Each lattice node **r** is connected to its *b* nearest neighbors by unit vectors **c**_*i*_, *i* = 1, …, *b*, called velocity channels. The total number of channels per node is defined by *K* ≥ *b*, where *K* − *b* is an arbitrary number of channels with zero velocity, called rest channels. Each channel can be occupied by at most one cell at a time. We consider a tumor population of two mutually exclusive cell phenotypes, moving (*m*) and resting (*r*). Moving cells reside on the velocity channels, indexed by *i* = 1, …, *b*, while resting cells are located within the rest channels, indexed by *i* = *b* + 1, …, *K*, of the lattice. The total number of cells at time *k* and node **r** is given by *n*(**r**, *k*) = *n*_*m*_(**r**, *k*) + *n*_*r*_(**r**, *k*), where *n*_*m*_ and *n*_*r*_ denote the moving and resting cell numbers, respectively. The parameter *K* is a local cell number bound. This constraint is imposed, since the maximal cell number in a given volume is limited in a biological tissue. Notice that *K* corresponds to a carrying capacity density and thereby accounts for cell crowding effects.

The time evolution of our model is defined by the following rules:

(R1) cells of both phenotypes die with probability *r*_*d*_,
(R2) resting cells proliferate with probability *r*_*b*_ unless the local carrying capacity is reached, i.e. all rest channels are occupied,
(R3) cells may change their phenotype from moving to resting (respectively, from resting to moving) with probability *r*_*s*_ (respectively, 1 − *r*_*s*_),
(R4) moving cells perform independent random walks.

**Fig 1:**
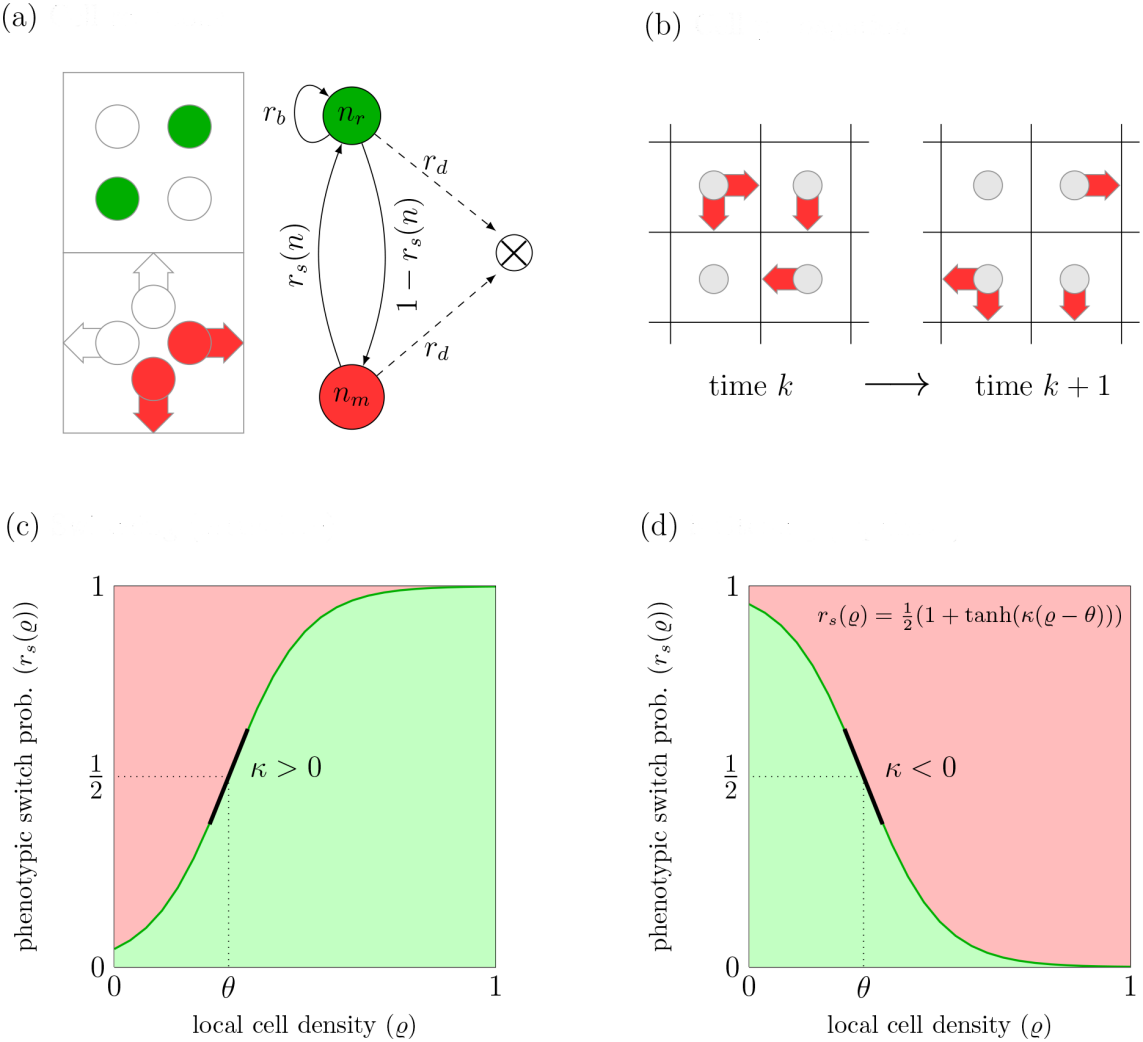
Cellular automaton model dynamics arise from repeated application of cell reactions and cell migration. (a) (left) Local state space at a given node is divided into rest channels and velocity channels. The cells in the rest channels, marked in green, are of proliferative phenotype. The cells in the velocity channels, marked in red, have the migratory phenotype. White channels denote absence of tumor cells. (right) Schematic illustration of model reactions. (b) Example of application of the cell propagation operator in the CA model. Cells in the velocity channels before and after a propagation step; red arrows denote the presence of a cell in the respective velocity channel. (c) and (d) Phenotypic plasticity in the CA model. Schematic illustration of the phenotypic switch probability *r*_*s*_ which depends on the cell density ϱ in the microenvironment. The sign of the phenotypic switch parameter *κ* determines the dynamic regime, *κ* > 0 results in attractive behavior (c) while repulsive behavior arises for *κ* < 0 (d).

In the LGCA, rules (R1)-(R4) are realized by applying three operators: A cell reaction operator changes the local cell numbers *n*_*r*_, *n*_*m*_ on each node according to (R1)-(R3). A reorientation operator randomly shuffles the configuration within the velocity channels at each node. By applying a propagation operator, moving cells are shifted one lattice unit in directions determined by their velocities. Both reorientation and propagation steps define cell movement, (R4). At each discrete time point *k*, the composition of the three operators is applied independently at every node on the lattice to compute the configuration at time *k* + 1, see Fig 1(a) and 1(b) and S1 Text.

We hypothesize that the phenotypic switch between proliferative and migratory cell behavior depends on the local cell density. We regard local cell density as the result of tumor cell interactions with extracellular matrix components, chemical cues and other stromal cells. Therefore, we model all these effects by means of their impact on cell density. We do not aim to reproduce the switch process in all intracellular detail. Rather we coarse-grain the intracellular details into stochastic cell-based rules that allow for an analytically tractable model to provide a basic understanding of the underlying dynamics. Since it is not known how the phenotypic switch depends on cell density, we decide for the most simple form which is monotonous dependence. Then, two complementary types of plasticity can be distinguished: *attraction* towards or *repulsion* from highly populated areas. In the attraction case, cell motility decreases with local cell density, so that proliferation is favored in densely populated areas. In the repulsion case, cells tend to escape from highly populated regions, that is cell motility increases with local cell density, and proliferation is favored in sparsely populated areas. The switch probability *r_s_*(*ϱ*) (respectively 1 − *r_s_*(*ϱ*)) that a moving cell becomes resting (or a resting cell becomes moving) is modeled as a sigmoidal shaped function *r*_*s*_:[0, 1] → (0,1) that depends on the cell density *ϱ* = *n/K* at the given node and two parameters *κ* ∈ ℝ and *θ* ∈ (0,1),

$$r_{s}\left(\varrho \right)=\frac{1}{2}\left(1+\tanh\left(\kappa \left(\varrho -\theta \right)\right)\right),\;\varrho \in \left[0,1\right].$$ (1)

The absolute value of *κ* specifies the intensity of the switch’ density dependence while its sign determines whether the attraction case (*κ* > 0) or the repulsion case (*κ* < 0) holds. Parameter *θ* represents the critical cell density value at which switching probabilities from one phenotype into the other are equal. We remark that the functional form of the switching function has been proposed in a previous study [19] where, however, tumor invasion behavior has been studied. A plot of the switching probabilities Eq (1) is given in Fig 1(c) and 1(d).

### Model analysis

We simulate the LGCA model on a two-dimensional square lattice (d=2, b=4) with 104 nodes. We explore the impact of the switch intensity *κ* and the switch position *θ* on the persistence of a growing tumor population. To this end, we investigate the total population growth rates in the (*κ*, *θ*)-parameter space and identify the parameter regimes for population survival and extinction. Proliferation and death probabilities are chosen such that 0 < *r*_*d*_ ≪ *r*_*b*_ ≪ 1. The initial model condition reflects a biological situation where the tumor is small and spatially constrained. At the initial time a fixed number of moving and resting cells per node is placed in a predefined radius from the lattice center. In the simulations, the initial cell density is varied by changing the percentage of occupied nodes within this radius. The results do not depend on the fixed initial radius (simulations not shown) but on the initial cell density.

## Results

### Emergent population growth dynamics

**Fig 2:**
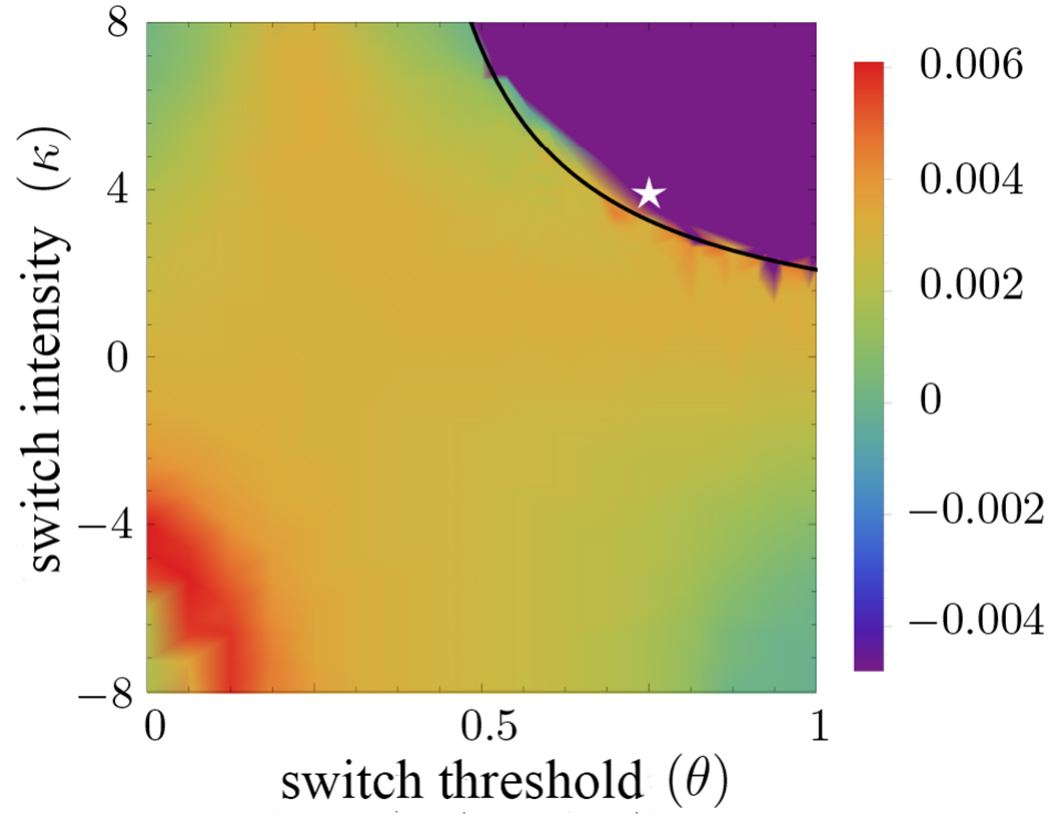
Total population growth rate of the resting cell population depends on the phenotypic switch parameters *κ* and *θ*. Initially, in a radius of 10 [unit length] from the center one rest and one velocity channel per node are occupied. Parameters are *r*_*b*_ = 0.2, *r*_*d*_ = 0.01, *K* = 8. Simulations are performed for 1000 iteration steps steps. The color indicates the total population growth rate of the resting cell population. The dark purple region denotes population extinction. The black curve is given by the equality *r_s_*(*ϱ*) = *r_d_/r_b_*, with *ϱ* = 0.25. The inequality derived in Eq (3) approximates the (*θ*, *κ*)-parameter region for which population extinction is observed. The white star represents the specific (*κ*, *θ*)-values for which subsequent analysis of the frequency of population extinction in Fig 3 is performed.

Fig 2 gives an overview of the observed cell population dynamics. In the repulsive case (*κ* < 0), the population always persists, regardless of the initial population density. In the attractive case (*κ* > 0), either survival or extinction may be observed, where the particular behavior is dependent on the specific values of *κ* and *θ*.

**Fig 3:**
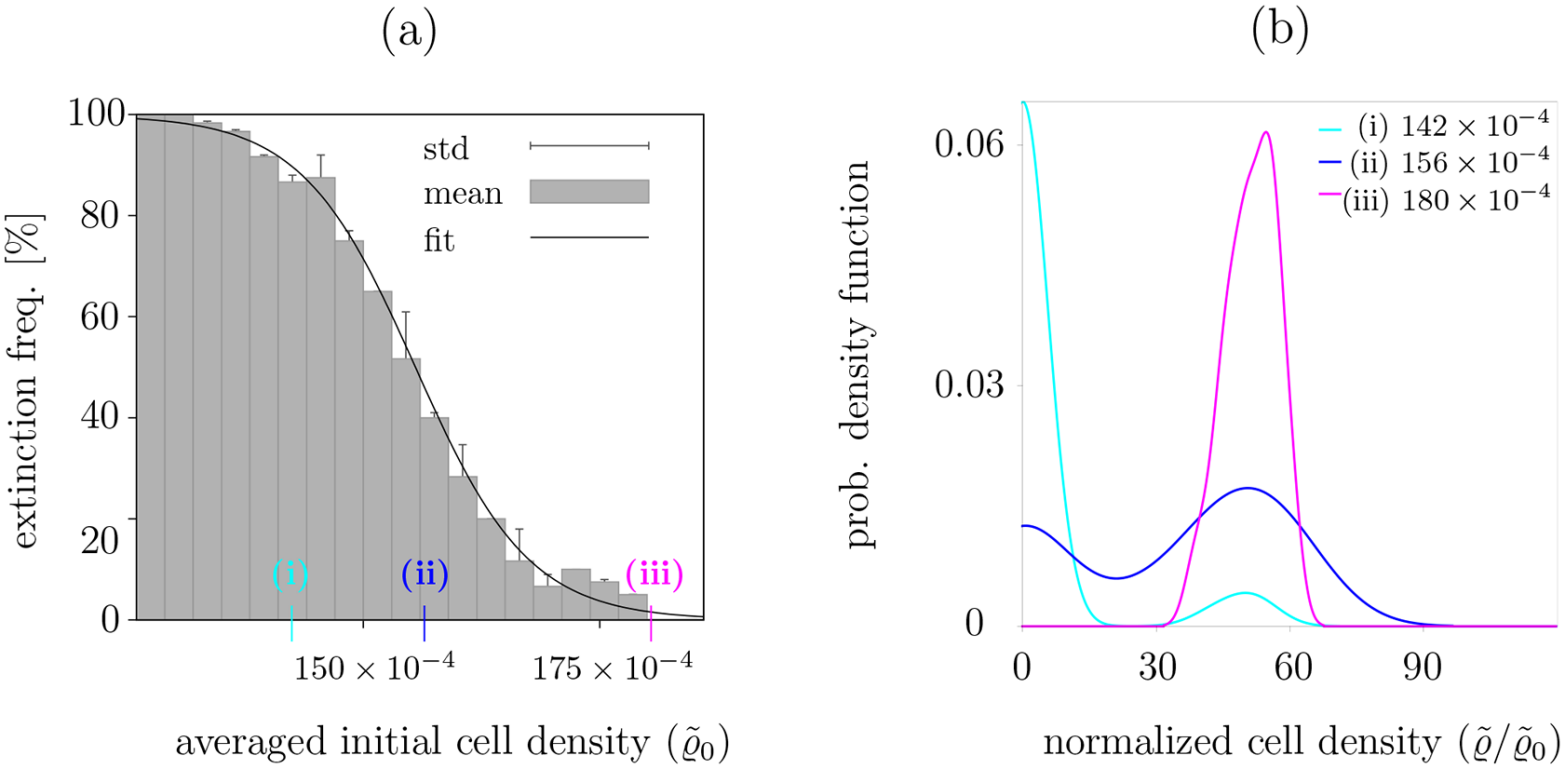
Frequency of population extinction in the CA model depends on the initial population size. (a) Stochastic fluctuations lead to extinction or growth. The figure shows the frequency of extinction events depending on averaged cell density $\tilde{\varrho }=|ℒ{|}^{-1}\sum_{r\in ℒ}\varrho (r)$. (b) Probability density function (derived by kernel density estimates, see S1 Text) of the averaged cell density after 5000 LGCA time steps for three different initial population densities. For each initial configuration 40 simulation runs are performed. Model parameters are *κ* = 4.4, *θ* = 0.75, *r*_*b*_ = 0.2, *r*_*d*_ = 0.01, *K* = 8. Cases (i), (ii) and (iii) refer to different initial population densities, which show bimodal (case (i) and (ii)) or unimodal (case (iii)) stationary size distributions.

Further, we investigate the survival of populations in the attractive case. For a wide range of different initial population densities, we record the frequency of extinction events. Sufficiently long simulation runs are performed to ensure that survival, when observed, is not a transient dynamical behavior (see S1 and S2 Videos). The results depicted in Fig 3(a) show that the smaller the initial population density the higher the probability of population extinction. Above a critical initial population density, the population always persists. Additionally, we record the population size distributions after a certain number of time steps for different initial cell densities, see Fig 3(b). One observes that low-density initial populations in the critical regime show bimodal stationary size distribution, indicating the possibility of either population extinction or persistence.

**Fig 4:**
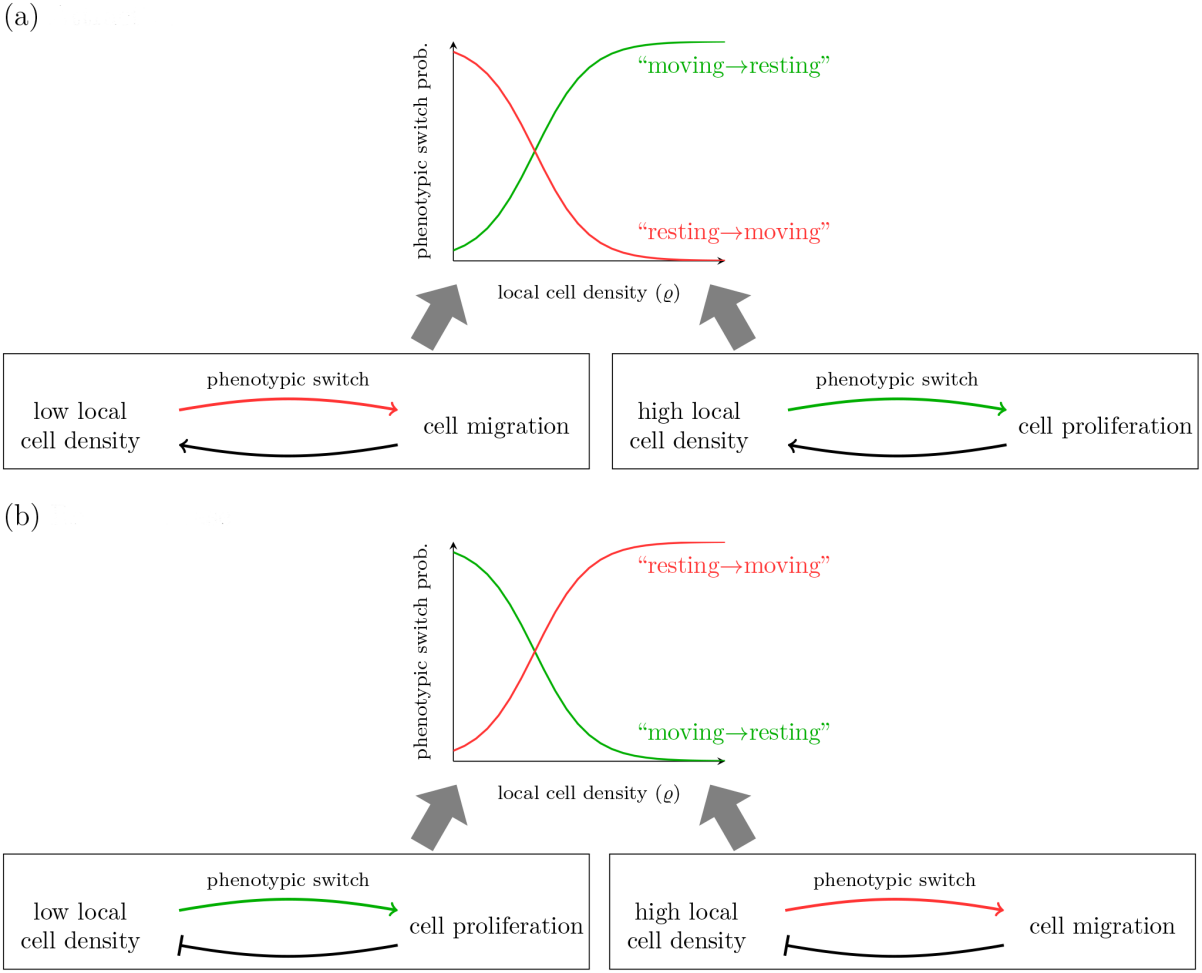
Sketch of the feedback mechanisms on single cell behavior (proliferative or migratory) for the different types of phenotypic plasticity (attractive or repulsive). (a) In the attractive case, the positive feedback between cell proliferation and high local cell density results in population persistence. However, positive feedback between cell migration and low local cell density might lead to population extinction if cell death overbalances replication. (b) In the repulsive case, the negative feedback mechanism on the cell scale leads to a balance of the population growth.

The observed emergent population behavior can be understood intuitively by considering the feedback mechanisms on the cell scale, in particular the regulation of proliferative or migratory behavior, for the different types of phenotypic plasticity (attractive or repulsive), see Fig 4. In the repulsion case (*κ* < 0), increasing local cell density has a negative feedback on proliferation. In a sparsely populated environment, cells are predominantly resting. In this case, when cell replication takes place, the resulting increase in local cell density triggers the switch to a migratory phenotype. Migration of cells decreases local cell density which again triggers the switch towards the resting cell phenotype. As a consequence, proliferation and migration phases alternate, and the population always persists. In the attraction case (*κ* > 0), increasing local cell density has a positive feedback on proliferation. Accordingly, cell proliferation leads to increased cell density which implies further proliferation. On the other hand, migration of cells locally decreases cell density that leads to more migratory cells. If the portion of resting cells in sparse environment is large enough, cell replication dominates cell death. Thus, the positive feedback on proliferation might result in population growth. However, if cells in sparse environment almost exclusively migrate, they eventually die, and the result is population *extinction*.

### Phenotypic plasticity gives rise to an Allee effect

The feedback mechanisms described above are just an intuitive picture of the emergent population dynamics. To better understand the connection between individual cell scale and the population properties, we derive a mean-field description of our model. It facilitates to link the microscopic interactions at the cellular level to effects that take place at the macroscopic scale. In particular, it allows us to analytically investigate the existence of an extinction threshold. The LGCA model is composed of a birth-death process describing the single-node cell reactions and a cell-movement process which describes the exchange of cells between neighboring nodes. Therefore, we derive mean-field approximations for each process separately first and then combine the resulting descriptions into a partial differential equation (PDE) for the whole tumor growth process. We then show that the resulting PDE, which exhibits a nonlinear diffusion term, has a kinetic term which is of a bistable nature in a suitable parameter range. Bistability is characterized by the presence of two stable steady states; one corresponds to the situation where the tumor dies out and the other one where the tumor persists. Bistable dynamics is significantly different from monostable dynamics, where only one stable steady state exists which corresponds to the situation where the tumor always persists. It is important to stress that the bistable behavior, which is already observed in the LGCA model, is preserved after the mean-field approximation thereof, as shown by the form of the kinetic term appearing on the corresponding PDE to be derived below.

If cell migration is neglected in the LGCA dynamics, the deterministic net changes occurring in the cell density of a given node between two consecutive times *k* and *k*+1 are determined by cell reactions only. Scaling time and transition rates such that the microscopic time *k* corresponds to the macroscopic time *t* = *τk*, *τ* ≪ 1, one obtains two ordinary differential equations for the migratory and proliferative cell density, respectively. However, such a system is hard to analyze analytically because of non-linearities which arise from the phenotypic switching. In order to facilitate analytical treatment, like bifurcation analysis, we assume that the switch dynamics is much faster than cell number changes due to proliferation and death. Furthermore, we consider the system to be in equilibrium with respect to the switching dynamics. Hence, for low cell density, the fractions of resting and moving cells are given by *r*_*s*_(*ρ*) and 1 − *r*_*s*_(*ρ*), respectively (see S1 Text). The overall macroscopic growth term of the LGCA model can then be approximated by

$$F\left(\rho \right)=R_{b}r_{s}\left(\rho \right)\rho \;(1-\rho )-R_{d}\rho ,$$ (2)

with *ρ*: = *ρ*_*m*_ + *ρ*_*r*_, where *ρ*_*m*_ and *ρ*_*r*_ is the mean cell density of moving and resting cells, respectively, at a given position. The parameters *R*_*b*_ and *R*_*d*_ relate the models’ proliferation and death parameters, *r*_*b*_ and *r*_*d*_, to the corresponding real time step length *τ*. If the average cell cycle time of a cell is given by *T*_*b*_ and the average life time of a cell by *T*_*d*_, then *R*_*b*_ = 1/*T*_*b*_ ≈ *r*_*b*_/*τ* and *R*_*d*_ = 1/*T*_*d*_ ≈ *r*_*d*_/*τ* (see S1 Text).

Stability analysis of the macroscopic net growth term *F*(*ρ*) shows that the behavior depends mainly on the type of phenotypic plasticity, attractive (*κ* > 0) or repulsive (*κ* < 0) (see S1 Text and S1 Fig). More precisely, one finds that there are essentially two regimes, a monostable one for *κ* < 0 and those values of *κ* > 0 for which *r_s_*(*ϱ*)*r_b_* > *r_d_* for small density values ϱ, and a bistable one for *κ* > 0 and

$$r_{s}\left(\varrho \right)r_{b}<r_{d}.$$ (3)

**Fig 5:**
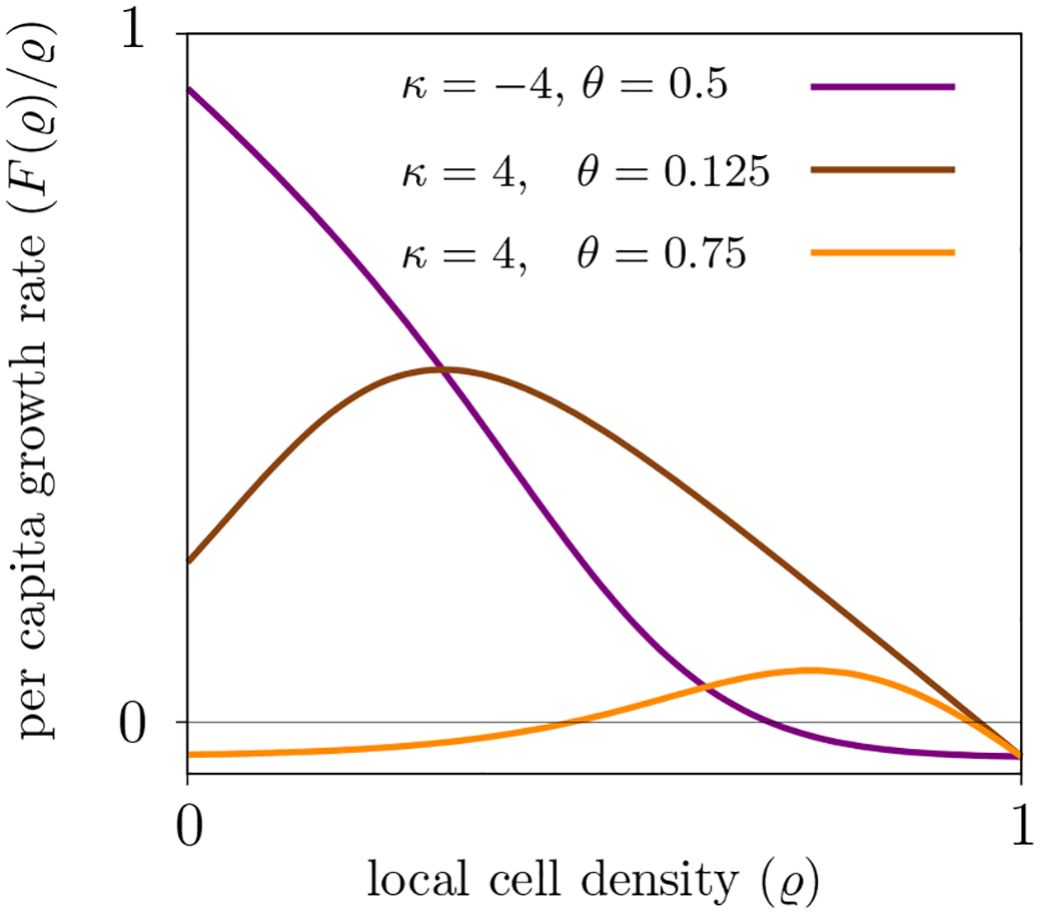
Per capita growth rate of the mean-field description Eq (2) depends on the type of phenotypic plasticity. In the repulsion case (*κ* < 0), the per-capita growth rate for small densities is always positive (purple line). In the attraction case (*κ* > 0), the per-capita growth rate is reduced at low density (brown line) and can even become negative (Allee effect, orange line). Model parameters are *r*_*b*_ = 0.2, *r*_*d*_ = 0.01, *K* = 8.

In the monostable regime, the cell density stabilizes at high density values where the exact location of the stable state is determined by the carrying capacity. In the bistable regime, additionally to the stable high-density state, the extinction state is stable. The critical region where *κ* > 0 and *r_s_*(*ϱ*)*r_b_* = *r_d_* for small density values ϱ is depicted in Fig 2 for *ϱ* = 0.25. This is in good agreement with the simulation results. The stability of the extinction state in the bistable regime is due to the fact that the per capita growth rate *F*(*ρ*)/*ρ* is negative for small density values, see Fig 5. Such negative density-dependence is termed Allee effect in the ecological literature and has been attributed strong impact on population persistence and invasion properties [31]. Here, the Allee effect emerges as a consequence of the phenotypic plasticity with respect to migratory and proliferative tumor phenotypes.

### Phenotypic plasticity leads to density-dependent migration

The mean-field approximation for the LGCA cell movement process, detailed in S1 Text, is also derived under the assumption that the switch dynamics are much faster than cell proliferation and death dynamics. Since then, for low cell densities, a density-dependent portion *r*_*s*_(*ρ*) of cells is in the resting state, the diffusion coefficient turns out to be density-dependent. The LGCA migration process is isotropic with respect to the principal lattice directions. Along any direction, the diffusion equation for the mean-field cell migration in the macroscopic limit is given by

$$\partial_{t}\rho =\partial_{x}\;(D\left(\rho \right)\partial_{x}\rho ),\;t\geq 0.$$ (4)

where the diffusion coefficient satisfies

$$D\left(\rho \right)=D\;(\frac{1-r_{s}\left(0\right)}{2}-r_{s}^{\prime }\left(0\right)\rho -\frac{3}{2}r_{s}^{\prime \prime }\left(0\right)\rho^{2}),\;\rho \ll 1,$$ (5)

with *D* being the diffusive scaling constant that is related to the single cell motility.

Combining the mean-field descriptions for the cell reaction and the cell migration processes, we obtain a single partial differential equation (PDE),

$$\partial_{t}\rho =\partial_{x}\;(D\left(\rho \right)\partial_{x}\rho )+F\left(\rho \right),$$ (6)

where the macroscopic growth term is given in Eq (2) and the density-dependent diffusion coefficient is given in Eq (5).

Scalar reaction-diffusion equations such as Eq (6) are comparatively easier to analyze than the original LGCA model, particularly in the simplified case where the diffusion coefficient *D*(*ρ*) is constant and the kinetic term is either purely monostable or bistable. For instance, in any of these situations, the resulting equation admits a particular type of solutions, traveling waves, which have been used as a paradigm to describe tumor invasion [32]. As recalled in [33], semilinear equations admit front travelling waves of the form *u*(*x*, *t*) = *U*(*x* − *ct*) = *U*(*z*), where *U*(*z*) connects two steady states of the equation as *z* tends to ±∞. In the bistable case, the wave speed is uniquely determined and can be positive or negative, depending on the precise form of *F*(*ρ*). In contrast, in the monostable case, infinitely many (only positive) wave speeds are possible, all of which should satisfy an explicit lower bound [34, 35]. Here, we are not interested in exploring the existence of traveling waves for the highly nonlinear Eq (6), since such particular type of solutions cannot keep track of extinction phenomena as those stressed in this work. We rather wish to point out another important difference between the monostable and bistable cases, which has been thoroughly discussed in the case of linear diffusivity. Namely, in the monostable case, invasion cannot be stopped once it starts [36, 37], whereas solutions corresponding to sufficiently small initial populations may eventually become extinct in the bistable case [38]. This issue is, for nonlinear diffusion, an interesting target for further theoretical studies.

## Discussion

In this work, we studied the effect of plasticity between migratory and proliferative behavior on tumor growth by means of a cellular automaton model. The trigger for the phenotypic switch was assumed to depend on the microenvironment via the local cell density. We found that this migration-proliferation plasticity has dramatic consequences for tumor growth. Two parameter regions with respect to the migratory cell behavior can be distinguished where fundamentally different tumor growth dynamics at the tissue scale are observed. In one case, called repulsive regime here, the tumor cell population will inevitably grow. In the other case, called attractive regime, we identified conditions under which sufficiently small tumors die out and tumor growth is only observed if the tumor size is above a certain threshold. We revealed that the extinction behavior is a consequence of an emergent negative net cell growth rate at low cell densities, a phenomenon known as Allee effect in ecology.

Comparing to previous studies [14, 18, 19] that investigate the effect of migration-proliferation plasticity with respect to the late phase of invasion and tumor spread, here we focus on an early phase of tumor growth and in particular initiation and persistence. Tektonidis et al. [19] have shown that glioma invasion data can be explained by assuming that the switching rates increase with increasing cell density while constant switching rates failed. However, it is not ruled out in this study that other parameter combinations, which might correspond to the attractive or repulsive case in our model, fit the data equally well. Finally, the authors in [19] refer to *in vitro* invasion data of a high-grade glioma cell line that according to our current results are likely to correspond to the repulsive case.

The Allee effect seems to have been overlooked so far in the context of tumor growth and persistence. In our study, the Allee effect emerges from the specific regulation of the migration-proliferation plasticity at the cellular scale and has not been assumed *a priori*. Since the Allee effect has been shown in ecology to change optimal control decisions, costs of control and the estimation of the risk posed by potentially invasive species [31], we expect it to be critical for tumor growth control as well. We point out that the Allee effect observed here is actually stochastic, displayed by the discrete cellular automaton model. This means that it is not an artifact arising from the mean-field approximation where stochastic effects are averaged out. In fact, our model actually accounts for stochastic fluctuations that may be particularly relevant for small to moderate size populations, a situation that may be relevant for tumor initiation.

In our model, the microenvironmental influence on the phenotypic switch between moving and proliferative cell behaviors is incorporated through a local cell density dependence. This is a plausible assumption since further potential environmental influences such as nutrient and oxygen supply, molecular signal gradients or other cell-cell interactions are mediated through and correlate with the local cell density. Note that this reduction of the underlying complexity is not a drawback of the model but allows to reveal inherent organizational principles. We expect that important population features like persistence and extinction could still be observed if the dependence of plasticity on local density is resolved into a more precise dependence on particular cell-microenvironment interactions, a subject that we intend to address in the future.

The stochasticity of our model incorporates heterogeneity of the microenvironment in its simplest form. Studying the implications of heterogeneity was not the focus of the current investigation as it would increase the model complexity and limit analytical investigations. However, ecological studies show that in many situations environmental heterogeneity has minor effects in growth dynamics where the Allee effect is present [39, 40]. In the future, investigations are required to analyze the importance of heterogeneity for specific tumors.

The Allee effect might have further implications for tumor spread. Here, we derived a mean-field description for the tumor cell density which is given by a reaction-diffusion equation with density-dependent diffusion coefficient and a potentially bistable reaction term. The behavior of solutions of fully nonlinear equations as Eq (6) is less known, although preliminary theoretical results [41] indicate a wealth of possible behaviors, including standing waves, oscillatory front and monotonic front solutions. It therefore seems that the Allee effect has a significant impact on population dispersal, which may be particularly relevant in the case of tumor dissemination.

Recently, in some tumors, the existence of a migration-proliferation dichotomy has been questioned [42]. It has been shown that individual cells in human malignant melanoma and lung cancer cell lines exhibit either migratory or proliferative behavior, but on the population level, migration and proliferation occur simultaneously. Experimental limitations due to the used *in vitro* approaches do not lead to a conclusive picture yet. Our contribution with respect to this discussion consists of two points. First, we provide a theoretical model which might help to resolve the differences between observations at the cellular and the population level. Second, for tumors with an established migration-proliferation plasticity at the cellular level, we provide predictions on overall tumor growth.

Our results might shed some light on the interpretation of recent experiments on tumor progression. For cell lines cultured from low-grade tumors, characterized by low cell density and well-differentiated tumor cells, it has been observed that they have low chances of persistence and low reproducibility *in vivo* and *in vitro* [6, 12, 43, 44]. On the contrary, cell lines from high-grade tumors, which are characterized by increased cellular density and less differentiated cells, repeatedly persist. Until now, the underlying mechanisms that lead to such different behaviors are unclear. We conjecture that the behavior of low-grade tumors resembles the attractive regime in our model while high-grade tumors behave as in the repulsive model regime. We suggest that the emergence of an Allee effect in low-grade tumors explains the existence of subcritical populations with low persistence probabilities. In contrast, the high-grade tumor cells always persist. Thus, we propose that the progression to malignancy may result from altered adaption to the cellular microenvironment with respect to the regulation of cell migration and proliferation. Concretely, we conjecture the glioma progression from low-grade glioma tumors to high-grade secondary gliomas corresponds to the evolution of attractive to repulsive cell dynamics, i.e. change in the sign of parameter *κ*.

The theoretical findings in our study might also provide suggestions for the design of new tumor therapies. Standard tumor therapy, such as chemo- and radiotherapy, are directed towards controlling cell proliferation. However, a recent study demonstrated that neoadjuvant chemotherapy selects for more migratory phenotypes at the expense of proliferative ones [45]. Our study shows that a possible therapeutic approach for malignant tumors is to combine conventional therapies with adjuvant treatments that restore sufficient contact inhibition of cell migration (CIM). In our model, CIM relates to a situation where cell motility decreases with increasing local cell density (attractive regime). In this case, if CIM is enforced, our model predicts that a sufficiently small tumor may die out due to the intrinsic cell population dynamics. On the contrary, if cell motility increases with local cell density (repulsive regime), which corresponds to CIM downregulation, any tumor inevitably grows and recurrence cannot be prevented. It is well known that malignant tumor cells lose sensitivity to contact inhibition of migration [46, 47]. Our study suggests that this is not only a bystander effect but might be a key determinant of tumor’s fate. Further investigations are required for the experimental validation of our hypothesis.

## Supporting Information

S1 Text Supplementary information. The supplementary text contains information about: 1) LGCA model description; 2) scaling of the LGCA; 3) equilibrium state under fast switching assumption; 4) mean-field approximation of the LGCA dynamics; 5) kernel density estimation of the averaged cell density and 6) stability analysis of cell reaction mean-field equation.
(PDF)

S1 Fig Stability analysis of the kinetic growth equation (27) in the S1 Text. The colored lines represent the kinetic growth function *F*(*ϱ*) (28) in the S1 Text for different values of *κ* and *θ*. Zeros of the function *F*(*ϱ*) correspond to fixed points of the LGCA cell reaction mean-field equation (27) in the S1 Text. The sign of the slope of function *F*(*ϱ*) at zero reveals the stability behavior of the fixed point. (a) In the repulsive case (*κ* < 0), there are exactly two fixed points. Parameters are *κ* = −4 and *θ* = 0.25. (b) In the attractive case (*κ* > 0), three cases can be distinguished: (i) one fixed point (orange-red line, *κ* = 4, *θ* = 0.98), (ii) two fixed points (brown line, *κ* = 4, *θ* = 0.125) or (iii) three (orange line, *κ* = 4, *θ* = 0.75) fixed points.
(PDF)

S1 Video Population extinction in the LGCA go-or-grow model. Initially, in a radius of 10 nodes from the center, in each node one rest and one velocity channel are randomly occupied (threshold 0.25). *r*_*m*_ = 0.2, *r*_*d*_ = 0.01, *K* = 8, *θ* = 0.375 and *κ* = 1.1. (1000 simulation time steps)
(AVI)

S2 Video Population growth in the LGCA go-or-grow model. Initially, in a radius of 10 nodes from the center, in each node one rest and one velocity channel are randomly occupied (threshold 0.25). *r*_*m*_ = 0.2, *r*_*d*_ = 0.01, *K* = 8, *θ* = 0.375 and *κ* = 1.1. (1000 simulation time steps)
(AVI)
