# Supplementary material for: An Emerging Allee Effect Is Critical for Tumor Initiation and Persistence
Source: PLoS Comput Biol. 2015 Sep 3;11(9):e1004366. doi: 10.1371/journal.pcbi.1004366 (PMC4559422; doi:10.1371/journal.pcbi.1004366)
Supplement: S1 Text — The supplementary text contains information about: 1) LGCA model description; 2) scaling of the LGCA; 3) equilibrium state under fast switching assumption; 4) mean-field approximation of the LGCA dynamics; 5) kernel density estimation of the averaged cell density and 6) stability analysis of cell reaction mean-field equation. (PDF) [file pcbi.1004366.s001.pdf]

Supplementary Information

Symbol	Explanation
$\mathcal{L} \subset \mathbb{Z}^d$	d-dimensional regular square lattice
$\mathbf{r} \in \mathcal{L}$	node on the lattice
$\sigma \in \{m, r\}$	cell phenotype in the model (moving m and resting r)
$K \geq 2d$	capacity of each lattice node ($2d$ velocity channels and $K - 2d$ resting channels)
$n(\mathbf{r}, k) \in \{0, \dots, K\}$	cell number at a node $\mathbf{r}$ and time k
$\varrho(\mathbf{r}, k) = n(\mathbf{r}, k)/K$	cell density at a node $\mathbf{r}$ and time k ($0 \leq \varrho(\mathbf{r}, k) \leq 1$)
$\rho(\mathbf{r}, k) = \langle n(\mathbf{r}, k) \rangle / K$	mean cell density at a node $\mathbf{r}$ and time k ($0 \leq \rho(\mathbf{r}, k) \leq 1$)
$k \in \mathbb{N}$	automaton time step
r_d	probability that a cell dies
r_b	probability that a resting cell undergoes mitosis
r_s	probability that a moving cell changes to resting
$1 - r_s$	probability that a resting cell changes to moving
$\kappa \in \mathbb{R}$	intensity of the phenotypic switch
$\theta \in (0, 1)$	critical cell density value at which the probabilities to switch from resting to moving and vice versa are equal
$\mathrm{P}(\cdot)$	probability
T_b	average cell cycle time
R_b	birth rate ($T_b = 1/R_b$)
T_d	average life time of a cell
R_d	death rate ($T_d = 1/R_d$)

Table 1. List of symbols

1 LGCA model description

Our LGCA is defined on a 2-dimensional square lattice $\mathcal{L} \subset \mathbb{Z}^2$. To every lattice node $\mathbf{r} \in \mathcal{L}$, velocity channels $(\mathbf{r}, \mathbf{c}_i)$, $i = 1, \dots, b$, are associated. The parameter b defines the number of nearest neighbors on the lattice. Here, we choose $b = 4$ and $c_i \in \{(1,0), (0,1), (-1,0), (0,-1)\}$. In addition, a fixed number of rest channels, $(\mathbf{r}, \mathbf{c}_i)$, $i = b+1, \dots, K$ with $c_i = \{(0,0)\}$ is introduced. The LGCA imposes an exclusion principle on channel occupation, i.e. at any time at most one cell is allowed in each channel at every lattice node. Thus K defines the maximal

capacity of cells per node. We represent the healthy cells by empty channels and model explicitly two tumor cell phenotypes, denoted by $\sigma \in \{m, r\}$: moving (m) and resting (r) tumor cells. The occupation numbers at time k, $\eta_i(\mathbf{r}, k)$, $i = 1, \ldots, K$, are random Boolean variables that indicate the presence ($\eta_i = 1$) or absence ($\eta_i = 0$) of a tumor cell in channel $(\mathbf{r}, \mathbf{c}_i)$. The local configuration of cells at a node $\mathbf{r}$ at time k is described by a vector $\boldsymbol{\eta}(\mathbf{r}, k) = (\eta_1(\mathbf{r}, k), \ldots, \eta_K(\mathbf{r}, k)) \in \{0, 1\}^K$. The total number of tumor cells at a node $\mathbf{r}$ and time k is defined by

$$n(\mathbf{r}, k) = n_m(\mathbf{r}, k) + n_r(\mathbf{r}, k) = \sum_{i=1}^{b} \eta_i(\mathbf{r}, k) + \sum_{i=b+1}^{K} \eta_i(\mathbf{r}, k),$$

where $n_m(\mathbf{r}, k)$ and $n_r(\mathbf{r}, k)$ are the number of moving and resting cells at a node $\mathbf{r}$ at time k, respectively.

The dynamics of our LGCA arise from the repeated application of three operators: **propagation** ($\mathcal{P}$), **reorientation** ($\mathcal{O}$) and **cell reactions** ($\mathcal{R}$). The propagation and reorientation operators define cell movement, whereas the cell reaction operator changes the local number of cells on a node. The composition $\mathcal{R} \circ \mathcal{O} \circ \mathcal{P}$ of the three operators is applied independently at every node $\mathbf{r}$ of the lattice and at each time k to evaluate the configuration at time $k + 1$.

The cell reactions operator comprises cell death and cell proliferation where the latter is affected by the migration-proliferation dichotomy. In detail, we consider three stochastic processes: phenotypic switching ($\mathcal{S}$), cell death ($\mathcal{D}$) and proliferation($\mathcal{B}$). For the sake of simplicity, the interaction between cells is restricted to the node itself. Moreover, we consider interaction rules which are independent of the cells' distribution to channels and solely depend on the total number of cells $n(\mathbf{r}, k)$ within the node. During one automaton time step, the number of cells on a node $\mathbf{r}$ changes by subsequent application of a switch ($\mathcal{S}$), death ($\mathcal{D}$) and proliferation ($\mathcal{B}$) step,

$$n = (n_m, n_r) \xrightarrow{\mathcal{S}} (n'_m, n'_r) \xrightarrow{\mathcal{D}} (n''_m, n''_r) \xrightarrow{\mathcal{B}} (n''_m, n'''_r) = n^{\mathcal{R}}. \quad (1)$$

The precise update rules are described below. Since we assume that individual cells decide to switch, die or divide independently from each other, the corresponding transition probability for the cell number per node follows a binomial distribution.

- *Phenotypic switch ($\mathcal{S}$)*: Cells can either rest or move. The phenotypic switch depends on the local cell density[1] $\varrho = n/K$. Phenotypes are changed with probabilities $r_s(\varrho)$ and $1 - r_s(\varrho)$ that denote the probabilities of a moving cell to become resting and vice versa. Moving and resting populations can exchange cells if there is enough free space on the lattice. Two steps are independently performed:

  (1) Define $M_1 = \min(n_m, (K - b) - n_r)$ which is the potential number of cells that can switch from the moving to the resting phenotype, taking into account the effect of local volume exclusion. The transition probability that there are j_1 successful events

[1] Cell density is defined as number of cells per unit volume. Given a typical size of a tumor cell, V_{cell} (units mm^3), one can easily estimate the cell density ϱ at a lattice node $\varrho = n/(K \cdot V_{cell}) = n/V_{node}$, where n is the number of cells at a lattice node, K is the maximal node capacity and $V_{node} = K \cdot V_{cell}$ (units mm^3) is the size of each lattice node. Thus, cell density can be easily rescaled to cell number.

'moving→resting' is modeled by

$$P_{\mathcal{S}_1}((n_m, n_r) \to (n_m - j_1, n_r + j_1)) = \begin{cases} \binom{M_1}{j_1} r_s(\varrho)^{j_1} (1 - r_s(\varrho))^{M_1 - j_1} & \text{if } 0 \le j_1 \le M_1, \\ 0 & \text{otherwise.} \end{cases} \tag{2}$$

(2) Define $M_2 = \min(n_r, b - n_m)$ which is the potential number of cells that can switch from the resting to the moving phenotype, taking into account the effect of local volume exclusion. The transition probability that there are j_2 successful events 'resting→moving' is given by

$$P_{\mathcal{S}_2}((n_m, n_r) \to (n_m + j_2, n_r - j_2)) = \begin{cases} \binom{M_2}{j_2} (1 - r_s(\varrho))^{j_2} r_s(\varrho)^{M_2 - j_2} & \text{if } 0 \le j_2 \le M_2, \\ 0 & \text{otherwise.} \end{cases} \tag{3}$$

Finally, the difference between successful switches 'moving→resting' and 'resting→moving' is used to update the number of moving and resting cells so that $(n_m, n_r) \to (n_m - (j_1 - j_2), n_r + (j_1 - j_2)) = (n'_m, n'_r)$. Note that the switching process does not change the total number of cells but the ratio between moving and resting cells.

- *Cell death ($\mathcal{D}$)*: Each tumor cell is assumed to die with probability r_d, independently from the other cells. Thus, the probability that j_1 moving and j_2 resting cells die, starting with n' cells is

$$P_{\mathcal{D}}((n'_m, n'_r) \to (n'_m - j_1, n'_r - j_2)) = \begin{cases} \binom{n'}{j} r_d^j (1 - r_d)^{n' - j} & \text{if } j_1 \le n'_m \text{ and } j_2 \le n'_r,\ j = j_1 + j_2, \\ 0 & \text{otherwise.} \end{cases} \tag{4}$$

- *Cell proliferation ($\mathcal{B}$)*: Birth of cells is dictated by the migration-proliferation dichotomy, which means that cells are allowed to proliferate only when they rest, i.e. when they are positioned on a rest channel. In addition, there must be empty rest channels to place the offspring into. Therefore, $M = \min(n''_r, (K - b) - n''_r)$ defines the potential number of resting cells that are allowed to proliferate starting from n'' cells after the switch and death processes. The transition probability that there are j new cells after the proliferation step is then given by

$$P_{\mathcal{B}}((n''_m, n''_r) \to (n''_m, n''_r + j)) = \begin{cases} \binom{M}{j} r_b^j (1 - r_b)^{M - j} & \text{if } j' \le M, \\ 0 & \text{otherwise.} \end{cases} \tag{5}$$

Altogether changes occurring in the cell number of a given node between two consecutive times k and $k + 1$ can be encoded in a transition matrix P with

$$P = P_{\mathcal{S}} P_{\mathcal{D}} P_{\mathcal{B}}, \tag{6}$$

where the entries of the transition matrix of the switch process $P_{\mathcal{S}}$ is determined by (2) and (3), and the transition matrices $P_{\mathcal{D}}$ and $P_{\mathcal{B}}$ of the death and proliferation processes, respectively, are defined by (4) and (5).

Subsequent to the cell reactions step, a reorientation step takes place where cells are randomly redistributed to the channels, providing a new local cell configuration at each node. Because of the migration-proliferation dichotomy, only the cells of migratory phenotype move. Therefore, moving cells are distributed to the velocity channels, while resting cells are distributed to the resting channels, resulting in a configuration $\eta^{\mathcal{R}\circ\mathcal{O}}(\mathbf{r})$.

The final update step is the application of a propagation operator. Cells in the velocity channels, i.e. moving cells, are transported one lattice unit in directions determined by their velocities. The movement of cells in the propagation step is purely deterministic. The spatio-temporal automaton dynamics is therefore completely described by the microdynamical equation

$$\eta_i(\mathbf{r}+\mathbf{c}_i, k+1) - \eta_i(\mathbf{r}, k) = \eta_i^{\mathcal{R}\circ\mathcal{O}}(\mathbf{r}, k) - \eta_i(\mathbf{r}, k) \in \{-1, 0, 1\},\ \mathbf{r} \in \mathcal{L},\ i = 1, \dots, K,\ k = 0, 1, \dots, \tag{7}$$

where the change in the occupation numbers is -1 if a channel looses a cell, 0 if nothing is changed and 1 if a channel gains a cell, respectively.

2 Scaling of the LGCA

We have chosen a nondimensional scaling with unit lattice spacing and unit time scale $k \in \mathbb{N}$ in our LGCA simulation. Thus, cells residing at velocity channels move one lattice unit per unit time step. Our dimensionless simulations can easily be rescaled such that the temporal and spatial scales fit to specific applications. In our case, cell death and proliferation probabilities are related to the real-dimensional average life time T_d of a cell and the average cell cycle time T_b, respectively, by

$$r_d(\tau) = \frac{\tau}{T_d},\ r_b(\tau) = \frac{\tau}{T_b}, \tag{8}$$

where τ is a sufficiently small real-dimensional time step length. This scaling is chosen because of the following arguments, detailed for the case of cell death. Let $X_1, X_2, \dots$ be independent Bernoulli trials with possible outcomes "cell dies" ($X_i = 1$) and "cell survives" ($X_i = 0$). The corresponding probability distribution is given by $\mathrm{P}(X_i = 1) = r_d$. Let N be the number of trials up to the first time a cell dies, i.e. $N := \min\{n | X_1 = \dots X_{n-1} = 0, X_n = 1\}$. Then N is geometrically distributed such that $\mathrm{P}(N = n) = (1 - r_d)^{n-1} r_d$ with expected value $\mathbb{E}[N] = 1/r_d$. If τ is the real-dimensional time step length, the life time of a cell is given by τN. Hence, the average cell life time in the model is $T = \mathbb{E}[\tau N] = \tau / r_d$ which must be related to the real-dimensional average life time T_d.

Similar arguments hold for the real macroscopic time taken for a cell to proliferate (cell cycle time) and for a cell to change its phenotype. Please note that if the LGCA parameters r_s, r_d and r_b depend on time τ, the transition matrix of the cell reactions becomes time-dependent:

$$P = P(\tau) = P_{\mathcal{S}}(\tau) P_{\mathcal{D}}(\tau) P_{\mathcal{B}}(\tau). \tag{9}$$

Besides scaling the LGCA time intervals, also the lattice spacing can be adjusted. If the lattice spacing is ϵ, the mean-square displacement of a cell per time step τ in the LGCA is proportional to ϵ^2/τ. Thus, by scaling $\mathbf{x} = \epsilon \mathbf{r} \in \mathbb{R}$, the relationship between the LGCA motility and measured movement of cells is characterized by the diffusion constant $D = \epsilon^2/\tau$.

3 Equilibrium state under fast switching assumption

When deriving the mean-field equation, we assume that the switch dynamics is much faster than the cell number changes due to proliferation and death, that is $r_s, 1 - r_s \gg r_b, r_d$. Further, we consider the low density regime where the carrying-capacity effects can be neglected when considering the switching. Then, by time scale separation, the fractions $p_\sigma := n_\sigma / n$, $\sigma \in \{r, m\}$ of resting and moving cells, respectively, are in detailed balance with respect to the switch dynamics. This amounts to

$$\begin{aligned} p_r(1 - r_s) &= p_m r_s \\ &= (1 - p_r) r_s. \end{aligned}$$

Hence $p_r = r_s$ or, equivalently,

$$n_r = r_s n. \tag{10}$$

4 Mean-field approximation of the LGCA dynamics

The LGCA model is composed of a cell movement and a cell reaction process. In the following, we separate the two processes and derive mean-field approximations for each one. We show that the cell movement in the automaton model can be approximated by a degenerate diffusion term, while cell reaction in the automaton leads to a nonlinear reaction term in the resulting partial differential equation description.

4.1 Scaling limit of the cell reaction process

In the following, we are interested in the time evolution of the cell density and neglect any spatial dependence between nodes. We assume that the system is in equilibrium with respect to the switching (fast switching assumption, see section 3). We consider the Markov process $(N_k)_{k \geq 0}$ which describes the number of cells at a node $r \in \mathcal{L}$ in the discrete phase space $\{0, \ldots, K\}$. The transition matrix of this process, derived from (6) and (10), is given by $W = W_\mathcal{D} W_\mathcal{B}$, where

$$W_\mathcal{D}(i,j) \quad = \quad \begin{cases} \binom{i}{j}(1 - r_d)^j r_d^{i-j} & \text{if } j \leq i, \\ 0 & \text{otherwise} \end{cases} \tag{11}$$

and

$$W_\mathcal{B}(i,j) \quad = \quad \begin{cases} \binom{M}{r_s j - r_s i} r_b^{r_s j - r_s i} (1 - r_b)^{M - r_s j + r_s i} & \text{if } r_s j - r_s i \leq M = \min(r_s i, K - i), \\ 0 & \text{otherwise.} \end{cases} \tag{12}$$

Here, the minimum function $\min(x, K - x) \approx x(K - x)\frac{1}{K}$, $x \in (0, K)$. This approximation underestimates the minimum function but provides an intuitive explanation as it describes the fraction of cells that are able to proliferate times the fraction of space that can be filled. Please note that, for simplicity, we use a notation where the dependence of the switch function r_s from the node density ϱ is dropped.

The probability to be in state j after one time step is

$$\mathrm{P}_{k+1}(j) := \mathrm{P}(N_{k+1}=j) = \sum_{i=0}^{K} W(i,j)\mathrm{P}(N_k=i) = \sum_{i=0}^{K} W(i,j)\mathrm{P}_k(i)$$

and in matrix notation

$$\mathrm{P}_{k+1} = W\mathrm{P}_k.$$

The transition of a microscopic process to a macroscopic description requires a temporal scaling relation between the macroscopic and microscopic variables (see section 2 for details). We assume a small parameter $\tau > 0$ that scales the time variable $t = k\tau$ such that

$$\mathrm{P}_{t+\tau} = W(\tau)\mathrm{P}_t,$$

where now the transition matrix W depends on the real-dimensional time step size τ. Rearranging terms gives

$$\begin{aligned} \mathrm{P}_{t+\tau} - \mathrm{P}_t &= (W(\tau) - I)\mathrm{P}_t, \\ \frac{\mathrm{P}_{t+\tau} - \mathrm{P}_t}{\tau} &= \frac{W(\tau) - I}{\tau}\mathrm{P}_t. \end{aligned}$$

Then, for $\tau \downarrow 0$

$$\frac{\partial}{\partial t}\mathrm{P}_t = Q\mathrm{P}_t \tag{13}$$

with intensity matrix $Q := \lim_{\tau\downarrow 0} \frac{W(\tau)-I}{\tau} = \frac{\partial}{\partial\tau}W(\tau)\big|_{\tau=0}$. Since $W(\tau) = W_{\mathcal{D}}(\tau)W_{\mathcal{B}}(\tau)$ it follows that

$$\begin{aligned} Q = \frac{\partial}{\partial\tau}W(\tau)\bigg|_{\tau=0} &= \left[\left(\frac{\partial}{\partial\tau}W_{\mathcal{D}}(\tau)\right)W_{\mathcal{B}}(\tau) + W_{\mathcal{D}}(\tau)\left(\frac{\partial}{\partial\tau}W_{\mathcal{B}}(\tau)\right)\right]\bigg|_{\tau=0} \\ &= \frac{\partial}{\partial\tau}W_{\mathcal{D}}(\tau)\bigg|_{\tau=0} + \frac{\partial}{\partial\tau}W_{\mathcal{B}}(\tau)\bigg|_{\tau=0} \\ &= Q_{\mathcal{D}} + Q_{\mathcal{B}}, \end{aligned} \tag{14}$$

where $Q_{\mathcal{D}}$ and $Q_{\mathcal{B}}$ are the intensity matrices for the death and birth processes, respectively. A direct calculation gives

$$\begin{aligned} Q_{\mathcal{D}} = \lim_{\tau\downarrow 0}\frac{1}{\tau}W_{\mathcal{D}}(\tau;i,j) &= \lim_{\tau\downarrow 0}\frac{1}{\tau}\begin{cases} \binom{i}{j}(1-r_d(\tau))^j r_d(\tau)^{i-j} & \text{if } 0 \le j \le i, \\ 0 & \text{else,} \end{cases} \\ &\overset{(8)}{=} \lim_{\tau\downarrow 0}\frac{1}{\tau}\begin{cases} \binom{i}{j}\left(1-\frac{\tau}{T_d}\right)^j\left(\frac{\tau}{T_d}\right)^{i-j} & \text{if } 0 \le j \le i, \\ 0 & \text{otherwise,} \end{cases} \\ &= \lim_{\tau\downarrow 0}\begin{cases} \frac{1}{T_d}\binom{i}{j}\left(1-\frac{\tau}{T_d}\right)^j\left(\frac{\tau}{T_d}\right)^{i-j-1} & \text{if } 0 \le j \le i, \\ 0 & \text{otherwise,} \end{cases} \\ &= \begin{cases} \frac{i}{T_d} & \text{if } j = i-1, \\ 0 & \text{otherwise} \end{cases} \end{aligned} \tag{15}$$

and similarly

$$Q_{\mathcal{B}} = \lim_{\tau \downarrow 0} \frac{1}{\tau} W_{\mathcal{B}}(\tau; i, j) = \begin{cases} \frac{1}{T_b} r_s i \left(1 - \frac{i}{K}\right) & \text{if } r_s j = r_s i - 1, \\ 0 & \text{otherwise.} \end{cases} \tag{16}$$

From the master equation (13), the temporal evolution of the mean cell density $\rho_t = \langle N_t \rangle / K$ is obtained by

$$\begin{aligned} \frac{\partial}{\partial t}\rho_t = \frac{1}{K}\sum_{j=0}^{K} j \frac{\partial}{\partial t}\mathrm{P}_t(j) \quad &= \quad \frac{1}{K}\sum_{j=0}^{K} j \sum_{i=0}^{K} Q(i,j)\mathrm{P}_t(i). &(17)\\ &= \quad \frac{1}{K}\sum_{i=0}^{K} \mathrm{P}_t(i) \sum_{j=0}^{K} jQ(i,j) \\ &\overset{(14)}{=} \quad \frac{1}{K}\sum_{i=0}^{K} \mathrm{P}_t(i) \sum_{j=0}^{K} jQ_{\mathcal{D}}(i,j) + \frac{1}{K}\sum_{i=0}^{K} \mathrm{P}_t(i) \sum_{j=0}^{K} jQ_{\mathcal{B}}(i,j) \\ &\overset{(15),(16)}{=} \quad \frac{1}{K}\frac{1}{T_d}\sum_{i=0}^{K}(-i)\mathrm{P}_t(i) + \frac{1}{K}\frac{1}{T_b}\sum_{i=0}^{K} r_s i \left(1 - \frac{i}{K}\right)\mathrm{P}_t(i) \\ &= \quad -\frac{1}{T_d}\rho + r_s\frac{1}{T_b}\rho - r_s\frac{1}{K}\frac{1}{T_b}\sum_{i=0}^{K} i^2 \mathrm{P}_t(i). &(18) \end{aligned}$$

Assuming that $\mathrm{E}[N_t^2] = \mathrm{E}[N_t]^2$, which holds if the variance of N_t is small, for instance if K is large, we finally arrive at a macroscopic description of the LGCA birth-death process

$$\frac{\partial}{\partial t}\rho_t = -R_d\rho + r_s(\rho)R_b\rho(1-\rho), \tag{19}$$

where $R_d = 1/T_d$ and $R_b = 1/T_b$ are the real death and proliferation rates, respectively, see section 2.

4.2 Scaling limit of the cell migration process

In the following, we consider the automaton cell migration process without cell reactions, under the fast switching assumption, see section 3. In our LGCA, the probability of a cell to jump from one node $\mathbf{r}$ to a neighboring node $\mathbf{r} + \mathbf{c}_i$ is equal to the probability of a cell residing in the respective velocity channel. In the isotropic case, cells within velocity channels are redistributed with probability $1/b$. To connect the discrete mechanism with a continuum model we consider the expected cell density of a node r at time k, defined by $\rho(r,k) = \langle \varrho(r,k) \rangle$, in the scaling limit when time step length and lattice spacing go to 0.

The change in average occupancy of a moving cell at site r during the next time step is given by

$$\rho(\mathbf{r}, k+1) = \sum_{i=1}^{b} \frac{1 - r_s(\rho(\mathbf{r}+\mathbf{c}_i, k))}{b}\rho(\mathbf{r}+\mathbf{c}_i, k) + r_s(\rho(\mathbf{r}, k))\rho(\mathbf{r}, k), \tag{20}$$

where the terms can be interpreted as the probability that a cell at site $\mathbf{r} + \mathbf{c}_i$, $i = 1, \ldots, b$, is moving to site $\mathbf{r}$ and the probability that a moving cell at site $\mathbf{r}$ does not attempt to leave the

site. In the following, for simplicity, we will analyze a one-dimensional situation where motility events only take place in the horizontal direction. This approach can easily be extended to higher dimensions, because of the model isotropy.

In one dimension, equation (20) reduces to

$$\rho(\mathbf{r},k+1)=\frac{1-r_s(\rho(\mathbf{r}-1,k))}{2}\rho(\mathbf{r}-1,k)+\frac{1-r_s(\rho(\mathbf{r}+1,k))}{2}\rho(\mathbf{r}+1,k)+r_s(\rho(\mathbf{r},k))\rho(\mathbf{r},k), \tag{21}$$

where the terms correspond to the probability that a cell at site $\mathbf{r}-1$ is moving and moves to the right, the probability that a a cell at site $\mathbf{r}+1$ is moving and moves to the left and the probability that a moving cell at site $\mathbf{r}$ does not attempt to leave the site. Approximating $r_s(\rho)$ around $\rho \ll 1$ such that $r_s(\rho) = r_s(0) + r_s'(0)\rho + r_s''(0)\rho^2 + \mathcal{O}(\rho^3)$, equation (21) becomes

$$\begin{aligned}\rho(\mathbf{r},k+1)&=\frac{1}{2}\left(\rho(\mathbf{r}+1,k)+\rho(\mathbf{r}-1,k)\right)-\frac{1}{2}r_s(0)(\rho(\mathbf{r}+1,k)+\rho(\mathbf{r}-1,k))\\&-\frac{1}{2}r_s'(0)(\rho^2(\mathbf{r}+1,k)+\rho^2(\mathbf{r}-1,k))-\frac{1}{2}r_s''(0)(\rho^3(\mathbf{r}+1,k)+\rho^3(\mathbf{r}-1,k))\\&+r_s(0)\rho(\mathbf{r},k)+r_s'(0)\rho^2(\mathbf{r},k)+r_s''(0)\rho^3(\mathbf{r},k).\end{aligned} \tag{22}$$

To convert the finite difference equation (22) into a continuous macroscopic partial differential equation (PDE), we identify the average density $\rho(\mathbf{r},k)$ by its continuous counterpart $\rho(\mathbf{x},t)$, where $\mathbf{x} = \mathbf{r}\epsilon \in \mathbb{R}$ and $t = k\tau \in \mathbb{R}_+$, with $\epsilon, \tau \in \mathbb{R}_+$, which gives

$$\begin{aligned}\rho(\mathbf{x},t+\tau)&=\frac{1}{2}\left(\rho(\mathbf{x}+\epsilon,t)+\rho(\mathbf{x}-\epsilon,t)\right)-\frac{1}{2}r_s(0)(\rho(\mathbf{x}+\epsilon,t)+\rho(\mathbf{x}-\epsilon,t))\\&-\frac{1}{2}r_s'(0)(\rho^2(\mathbf{x}+\epsilon,t)+\rho^2(\mathbf{x}-\epsilon,t))-\frac{1}{2}r_s''(0)(\rho^3(\mathbf{x}+\epsilon,t)+\rho^3(\mathbf{x}-\epsilon,t))\\&+r_s(0)\rho(\mathbf{x},t)+r_s'(0)\rho^2(\mathbf{x},t)+r_s''(0)\rho^3(\mathbf{x},t).\end{aligned} \tag{23}$$

Expanding all terms in (23) in truncated Taylor series in powers of ϵ and τ up to second order gives

$$\begin{aligned}\rho+\tau\partial_t\rho+\frac{\tau^2}{2}\partial_{tt}\rho=\rho&+\frac{\epsilon^2}{2}\partial_{xx}\rho-r_s(0)\rho-\frac{r_s(0)\epsilon^2}{2}\partial_{xx}\rho-r_s'(0)\rho^2-r_s'(0)\epsilon^2(\partial_x\rho)^2\\&-r_s'(0)\rho\epsilon^2\partial_{xx}\rho-\frac{r_s'(0)\epsilon^4}{2}(\partial_{xx}\rho)^2-r_s''(0)\rho^3-3r_s''(0)\rho\epsilon^2(\partial_x\rho)^2\\&-\frac{3r_s''(0)\epsilon^2}{2}\rho^2\partial_{xx}\rho-\frac{3r_s''(0)\epsilon^4}{2}(\partial_x\rho)^2\partial_{xx}\rho-\frac{3r_s''(0)\epsilon^4}{4}\rho(\partial_{xx}\rho)^2\\&-\frac{r_s''(0)\epsilon^4}{2}(\partial_{xx}\rho)^3+r_s(0)\rho+r_s'(0)\rho^2+r_s''(0)\rho^3.\end{aligned} \tag{24}$$

To proceed, we consider the diffusive limit, i.e. $\epsilon \to 0$, $\tau \to 0$ and $\lim\limits_{\epsilon,\tau\to 0}\frac{\epsilon^2}{\tau} = \mathit{const} := D$. This

gives

$$
\begin{aligned}
\partial_t\rho &= \frac{D}{2} - \frac{D}{2}r_s(0)\partial_{xx}\rho - r_s'(0)D(\partial_x\rho)^2 - r_s'(0)D\rho\partial_{xx}\rho - 3r_s''(0)D\rho(\partial_x\rho)^2 - \frac{3D}{2}r_s''(0)\rho^2\partial_{xx}\rho \\
&= D\partial_{xx}\rho \underbrace{\left(\frac{1-r_s(0)}{2} - r_s'(0)\rho - \frac{3}{2}r_s''(0)\rho^2\right)}_{=:\mathcal{D}(\rho)} + D(\partial_x\rho)^2 \underbrace{\left(-r_s'(0) - 3r_s''(0)\rho\right)}_{=\mathcal{D}'(\rho)}.
\end{aligned}
\tag{25}
$$

Finally, the jump process of our LGCA can be approximated by a degenerate diffusion equation

$$\partial_t\rho = D\partial_x(\mathcal{D}(\rho)\partial_x\rho). \tag{26}$$

5 Kernel density estimation of the averaged cell density

The kernel density estimation, also known as Parzen-Rosenblatt density estimation, is a well known nonparametric way of estimating the probability density function underlying a finite set of observations [1, 2]. We use a built-in Mathematica function to estimate the kernel density of the averaged cell density. For details see the Wolfram Demonstrations Project `http://demonstrations.wolfram.com/KernelDensityEstimation/` and `http://reference.wolfram.com/mathematica/ref/SmoothKernelDistribution.html`.

6 Stability analysis of cell reaction mean-field equation

In the following, we analyze the stability behavior of the macroscopic growth equation (19) derived in section 4.1. Thus, let us consider the kinetic equation

$$\partial_t\rho = F(\rho), \tag{27}$$

where

$$F(\rho) = r_s(\rho)r_b\rho(1-\rho) - r_d\rho, \tag{28}$$

with positive constants r_b and r_d and

$$r_s(\varrho) = \frac{1}{2}(1 + \tanh(\kappa(\varrho - \theta))), \quad \rho \in [0,1],\ \kappa \in \mathbb{R},\ \theta \in (0,1). \tag{29}$$

Obviously, $F(0) = 0$ and $F(1) = -r_d < 0$. The first derivative of F with respect to ϱ is

$$F'(\varrho) = r_b\varrho(1-\varrho)r_s'(\varrho) + r_b r_s(\varrho)(1-2\varrho) - rd, \tag{30}$$

where $r_s'(\varrho) = \frac{1}{2}\kappa\,\mathrm{sech}^2(\kappa(\varrho - \theta))$. For the trivial fixed point $\rho_1 = 0$ of (27), it is

$$F'(0) = \frac{1}{2}r_b(1 - \tanh(\kappa\theta)) - r_d.$$

In order to identify fixed points of (27) and to determine their switch behavior, we will consider two conditions

$$\tanh(\kappa\theta) > 1 - 2\frac{r_d}{r_b} \tag{31}$$

and

$$\tanh(\kappa\theta) < 1 - 2\frac{r_d}{r_b}. \tag{32}$$

Lemma 1. The zero $\rho_1 = 0$ of $F(\varrho)$ is a stable equilibrium of (27) if condition (31) holds. Under condition (32), $\rho_1 = 0$ is an unstable equilibrium of (27).

Proof. Obviously, $\rho_1 = 0$ is a fixed point of (27). It is

$$F'(0) = \frac{1}{2}r_b(1 - \tanh(\kappa\theta)) - r_d < 0$$

if and only if $1 - 2\frac{r_d}{r_b} < \tanh(\kappa\theta)$. In contrast, $F'(0) > 0$ if and only if $1 - 2\frac{r_d}{r_b} > \tanh(\kappa\theta)$. Therefore, if condition (31) holds it is $F'(0) < 0$ and $\rho_1 = 0$ is stable. However, if condition (32) is satisfied, it is $F'(0) > 0$ and $\rho_1 = 0$ is unstable □ □

Lemma 2. Let $r_b < r_d$. Then $\rho_1 = 0$ is the only fixed point of (27).

Proof. For $\rho \in (0, 1]$, it is $(1 - \rho) \in [0, 1)$ and $r_s(\varrho) \in (0, 1)$. Thus

$$F(\rho) = \rho(\underbrace{r_b r_s(\rho)(1 - \rho)}_{\in[0,r_b]} - r_d) < 0$$

if and only if $r_b < r_d$. In this case, $\rho_1 = 0$ is the only zero of $F(\rho)$. □ □

Remark 1. For $r_b < r_d$, it is $1 - 2\frac{r_d}{r_b} < -1$. Then condition (31) is always satisfied if $\kappa < 0$, because in this case it is $\tanh(\kappa\theta) \in (-1, 0)$.

For $r_b > r_d$, we will consider the two cases $\kappa < 0$ (repulsive case) and $\kappa > 0$ (attractive case).

Lemma 3. Let $\kappa < 0$ and $r_b > r_d$. If condition (32) holds, one unstable fixed points $\rho_1 = 0$ and one stable fixed point $0 < \rho_2 < 1$ of (27) exist.

Proof. Under condition (32), it is $F'(0) > 0$ and F has exactly one maximum in $\rho_{\max}$, where $0 < \rho_{\max} < 1$. F is strictly monotonic increasing in $[0, \rho_{\max})$ and strictly decreasing in $[\rho_{\max}, 1]$. By the intermediate value theorem it exists one $\rho_2 \in (0, 1)$ with $F(\rho_2) = 0$ and $F'(\rho_2) < 0$. This ρ_2 is unique by the strict monotony of F. □ □

Lemma 4. Let $\kappa > 0$ and $r_b > r_d$. If condition (32) is satisfied, one unstable fixed points $\rho_1 = 0$ and one stable fixed point $0 < \rho_2 < 1$ of (27) exist.

Proof. For $\kappa > 0$ and under condition (32) it is $F'(0) > 0$ and F has one maximum in $\rho_{\max}$, where $0 < \rho_{\max} < 1$, and one minimum in $\rho_{\min} = 1$. F is strictly monotonic increasing in $[0, \rho_{\max})$ and strictly decreasing in $[\rho_{\max}, 1]$. By the intermediate value theorem, there exists a $\rho_2 \in (0, 1)$ with $F(\rho_2) = 0$ and $F'(\rho_2) < 0$. This ρ_2 is unique by the strict monotony of F. □ □

Lemma 5. Let $\kappa > 0$, $r_b > r_d$ and condition (31) be true. If $r_s(\rho)(1-\rho) < \frac{r_d}{r_b}$ for all $0 \leq \rho \leq 1$, then $\rho_1 = 0$ is the only fixed pint of (27). Otherwise, an unstable fixed point ρ_2 and two stable fixed points $\rho_1 = 0$, ρ_3 of (27) exist, with $0 < \rho_2 < \rho_3 < 1$.

Proof. For $\kappa > 0$ and under condition (31) it is $F'(0) < 0$. Moreover, F has one minimum in $\rho_{\min}$, where $0 < \rho_{\min} < 1$ and one maximum in $\rho_{\max}$, where $\rho_{\min} < \rho_{\max} < 1$. F is strictly monotonic decreasing in $[0, \rho_{\min}]$, strictly increasing in $(\rho_{\max}, \rho_{\max})$ and again strictly decreasing in $[\rho_{\max}, 1]$. If $(1-\rho)r_s(\rho) < \frac{r_d}{r_b}$ for all $0 \leq \rho \leq 1$, then $F(\rho) < 0$ for all $0 < \rho \leq 1$ and no other fixed point than the unstable $\rho_1 = 0$ exist. If, however, values of ρ exist for which $r_s(\rho)(1-\rho) > \frac{r_d}{r_b}$, then it exists $\rho^* \in (0,1)$ with $F(\rho^*) > 0$. By the intermediate value theorem it exists a ρ_2 and ρ_3, with $0 < \rho_2 < \rho^*$ and $\rho^* < \rho_3 < 1$, where $F(\rho_2) = 0$ and $F'(\rho_2) > 0$, and $F(\rho_3) = 0$ and $F'(\rho_3) < 0$. This ρ_2 and ρ_3 are unique by the strict monotony of F. □ □

Remark 2. For large values of θ and κ, it is $r_s(\rho)(1-\rho) < \frac{r_d}{r_b}$ for all $0 \leq \rho \leq 1$.

Remark 3. If, values of ρ exist for which $r_s(\rho)(1-\rho) > \frac{r_d}{r_b}$, then (27) shows bistable behavior and population extinction can be observed for small densities if (27)

$$r_s(\rho) < \frac{r_d}{r_b} \tag{33}$$

holds.

Finally, for $r_b > r_d$, we conclude that in the repulsive case ($\kappa < 0$), equation (27) has one unstable ($\rho_1 = 0$) and one stable ($0 < \rho_2 < 1$) fixed point. In the attractive case ($\kappa > 0$), three cases can be distinguished: (i) Equation (27) has one stable steady state if $\tanh(\kappa\theta) > 1 - 2\frac{r_d}{r_b}$ and $r_s(\rho)(1-\rho) < \frac{r_d}{r_b}$ for all $0 \leq \rho \leq 1$, (ii) one unstable ($\rho_1 = 0$) and one stable ($0 < \rho_2 < 1$) fixed point, (iii) one unstable ($0 < \rho_2 < 1$) and two stable ($\rho_1 = 0$, $\rho_2 < \rho_3 < 1$) fixed points. An illustration of the stability behavior under different parameter combinations is shown in Figure 7.

References

[1] Rosenblatt M (1959) Remarks on some nonparametric estimates of a density function. *Ann Math Stat* 27:832837.

[2] Parzen E (1962) On estimation of a probability density function and mode. *Ann Math Stat* 33(3):10651076.

7 Supporting Information Legends

Figure Legends

Figure 1. Stability analysis of the kinetic growth equation (27). The colored lines represent the kinetic growth function $F(\varrho)$ (28) for different values of κ and θ. Zeros of the function $F(\varrho)$ correspond to fixed points of the LGCA cell reaction mean-field equation (27). The sign of the slope of function $F(\varrho)$ at the zero reveals the stability behavior of the fixed point. (a) In the repulsive case ($\kappa < 0$), there are exactly two fixed points. Parameters are $\kappa = -4$ and $\theta = 0.25$. (b) In the attractive case ($\kappa > 0$), three cases can be distinguished: (i) one fixed point (orange-red line, $\kappa = 4$, $\theta = 0.98$), (ii) two fixed points (brown line, $\kappa = 4$, $\theta = 0.125$) or (iii) three (orange line, $\kappa = 4$, $\theta = 0.75$) fixed points.

Movie Legends

Movie S1. Population extinction in the LGCA go-or-grow model. Initially, in a radius of 10 from the center one rest and one velocity channel is randomly occupied (threshold 0.25). $r_m = 0.2$, $r_d = 0.01$, $K = 8$, $\theta = 0.375$ and $\kappa = 1.1$. (1000 simulation time steps)

Movie S2. Population growth in the LGCA go-or-grow model. Initially, in a radius of 10 from the center one rest and one velocity channel is randomly occupied (threshold 0.25). $r_m = 0.2$, $r_d = 0.01$, $K = 8$, $\theta = 0.375$ and $\kappa = 1.1$. (1000 simulation time steps)
