# Supplementary figures and images for: An Emerging Allee Effect Is Critical for Tumor Initiation and Persistence
Source: PLoS Comput Biol. 2015 Sep 3;11(9):e1004366. doi: 10.1371/journal.pcbi.1004366 (PMC4559422; doi:10.1371/journal.pcbi.1004366)

(a)

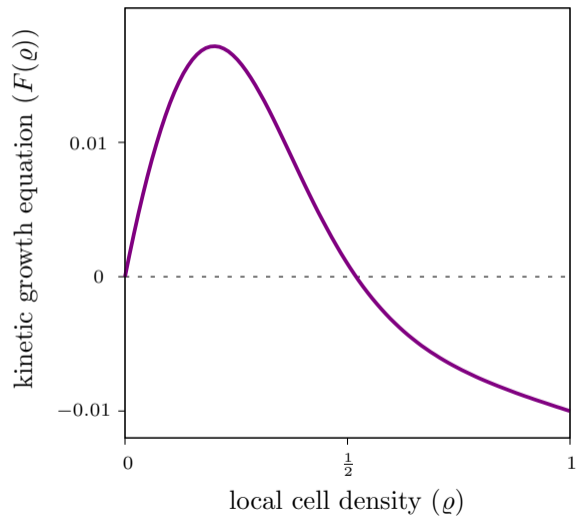

(b)

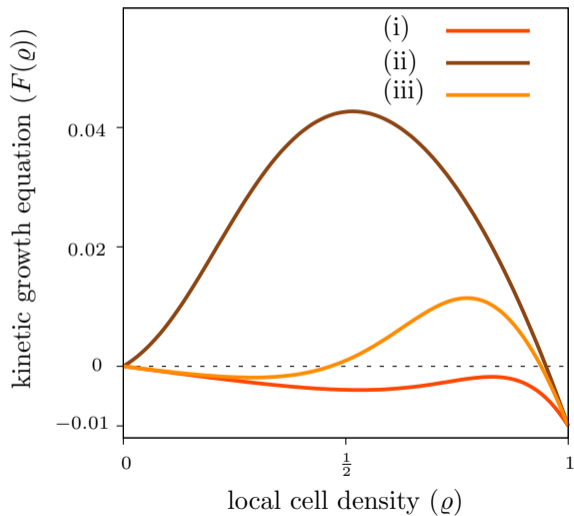

Supplement: S1 Fig — The colored lines represent the kinetic growth function F(ϱ) (28) in the S1 Text for different values of κ and θ. Zeros of the function F(ϱ) correspond to fixed points of the LGCA cell reaction mean-field equation (27) in the S1 Text. The sign of the slope of function F(ϱ) at zero reveals the stability behavior of the fixed point. (a) In the repulsive case (κ < 0), there are exactly two fixed points. Parameters are κ = −4 and θ = 0.25. (b) In the attractive case (κ > 0), three cases can be distinguished: (i) one fixed point (orange-red line, κ = 4, θ = 0.98), (ii) two fixed points (brown line, κ = 4, θ = 0.125) or (iii) three (orange line, κ = 4, θ = 0.75) fixed points. (PDF) [file pcbi.1004366.s002.pdf]
